# Effects of Printing Parameters on Properties of FDM 3D Printed Residue of Astragalus/Polylactic Acid Biomass Composites

**DOI:** 10.3390/molecules27217373

**Published:** 2022-10-30

**Authors:** Wangwang Yu, Jianan Shi, Liwei Sun, Wen Lei

**Affiliations:** 1 College of Science, Nanjing Forestry University, Nanjing 210037, China; 2 School of Mechanical Engineering, Nanjing Vocational University of Industry Technology, Nanjing 210023, China

**Keywords:** poly(lactic acid) (PLA), residue of Astragalus (ROA), composite, fused deposition modeling (FDM), 3D printing

## Abstract

In order to develop a new kind of filament material for the fused deposition modeling (FDM) 3D printing, the residue of Astragalus (ROA), one of the most important Chinese herbal medicines, and polylactic acid were chosen as the raw materials to FDM 3D print biomass composite specimens, the effects of the printing parameters on the properties of the specimens were investigated. The results indicated that the mechanical properties and thermal stability of the printed specimen were affected obviously by the parameters while the melting and crystallization behavior of the specimens were little affected. For the wettability, it was also little affected by the printing parameter except for the printing speed. Increasing the printing temperature and the filling density or reducing the printing speed and the layer thickness could improve both the mechanical properties and the thermal stability of the FDM 3D printed PLA/ROA composite specimen; reducing the deposition angle could also improve the mechanical properties while having little effect on the thermal stability of the specimen.

## 1. Introduction 

In recent years, three-dimensional (3D) printing technology has been developed rapidly in molding and manufacturing of materials and has attracted widespread attention; this technology has been used in a variety of fields, such as traffic engineering, civil engineering, biomedical engineering, aerospace, food industry, clothing, and architecture [1,2,3]. There are four main technologies of 3D printing, i.e., fused deposition modeling (FDM), stereo lithography (SL), selective laser sintering (SLS), and layered solid manufacturing (LSM). Among them, FDM is the most extensively used [1] because of its simplicity, flexible and rapid process, low cost, reliability, minimal waste, diversity of materials, and adaptability to new materials. 

Some polymer materials have been used in FDM, among which the application of polylactic acid (PLA) is the most common. PLA, a biodegradable polymer that comes from renewable resources, has many advantages, such as favorable mechanical properties, low elongation at break, low thermal expansion coefficient, biocompatibility, low toxicity, good melt processability, and reproducibility. Nevertheless, there are also some obvious defects in the properties of PLA which limit the further popularization and application in additive manufacturing technology such as lower impact properties, high brittleness, and poor thermal stability, so it is very necessary to do some research on the modification of PLA. Incorporation of abundant, cheap, and recyclable natural fiber into PLA is an effective method to remedy the defects and improve the properties of PLA. A series of studies have focused on the FDM 3D printing of natural fiber/PLA composites. The natural fibers involved include wood [4,5], flax [6,7], kenaf [8], ramie [9], jute [10], sugarcane bagasse [11], bamboo [12], rice straw [13], rice husk [4], energy grass [14], and so on. For FDM 3D printed natural fiber/PLA composite, its properties are decided to a great extent by the printing processes/parameters, thus the effect of the printing processes/parameters on the performance of FDM 3D printed specimen is currently being investigated by many researchers to promote the wider development of FDM [5,15]. Nadir Ayrilmis et al. [16] reported that water absorption and mechanical properties of wood/PLA composite materials produced by the FDM method were changed by the printing layer thickness, and that decreasing printing layer thickness resulted in the improved tensile and bending properties, and decreased water absorption of the samples. A. Le Duigou et al. [5] found that the mechanical properties of FDM 3D printed wood fiber/PLA composites strongly depended on the printing orientation, and also on the printing width. The Young’s modulus of the samples was much lower than in the compressed samples because of its microstructure with relatively high porosity (around 20%). Hao et al. [11] experimented sugarcane bagasse fiber infused into PLA for FDM 3D printing, exploring the effects of the printing orientation on the tensile performance of the printed composites; the final results showed that the fully oriented sample printed by method “parallel” had a better tensile strength. 

As one important part of natural fibers, the residue of Chinese herbal medicine has seldomly been reported to be used for FDM 3D technology. Traditionally, the residue is often disposed after drug component extraction by cooking, which leads to a great waste of natural fiber and some environment pollution. However, the incorporation of the residue of Chinese herbal medicine into polymer for 3D printing can not only integrate the advantages of light weight and corrosion resistance of polymer materials and low cost of natural fiber, but also the printed samples may have some other prominent properties, such as the medical effects on the environment and the special pleasant smell. The printed products may be applied as automotive interior trims, handicraft articles, home interior decoration board, and so on. As one of the most important Chinese herbal medicines, Astragalus has been widely planted in China; lots of the residue of Astragalus (ROA) is produced every year, and, as aforementioned, it is of great significance to explore the potential structural and economic performance of ROA for in the manufacturing of composites by FDM 3D technology. 

The focus of this article was to develop a new kind of filament material based on the composite of ROA and PLA, the emphasis was on the investigation of the effects of printing parameters on mechanical, thermal, and melting properties of the composites, additional emphasis was placed on the wettability of the composites. 

## 2. Materials and Methods

### 2.1. Materials and Reagents 

PLA (American Nature Works Co., 3052D, Minnetonka, MN, USA) was purchased from Shanghai Xingyun International Trade Co. Ltd., China (Shanghai, China); ROA, 120 mesh, was self-manufactured in our laboratory.

### 2.2. Production of FDM Filaments

In order to develop the filament by using ROA as much as possible, the mass ratio of ROA to PLA was first set as 15:85; however, it was found that the nozzle was often blocked, and the printing of the specimens could not be carried out smoothly. Then, the dosage of ROA was gradually reduced and, after a series of experiments, the mass ratio of ROA to PLA was finally selected as 11:89. 

**Figure 1:**
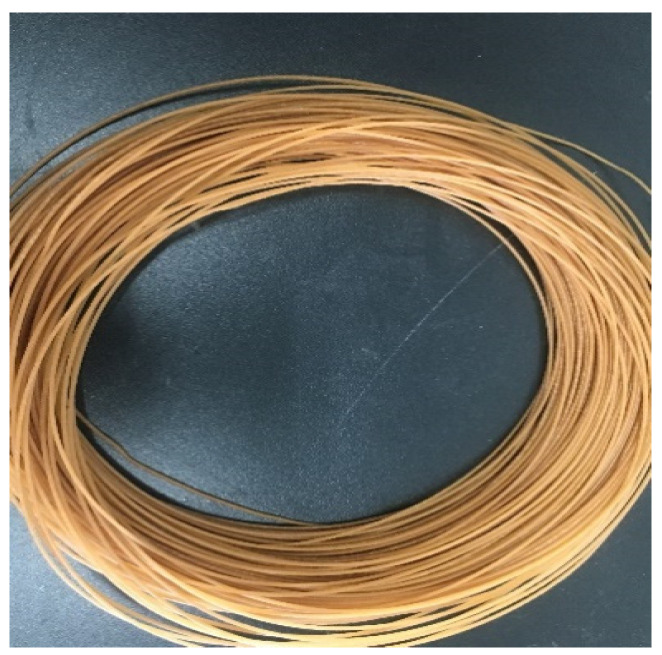
PLA/ROA composite filament.

ROA was dried before using. ROA and PLA were first mechanically mixed, then the mixture was pelletized using a twin-screw extruder (SHJ-20, Nanjing Giant Machinery Co. Ltd., Nanjing, China). The PLA/ROA composite filaments with a diameter of 1.75 ± 0.05 mm (Figure 1) were finally prepared using a twin-screw extruder (KS-HXY, Kunshan HUANXINYANG Electrical Equipment Co. Ltd., Suzhou, China) at the temperature of 170 °C to 190 °C from hopper to die.

### 2.3. Specimens Manufacturing 

**Figure 2:**
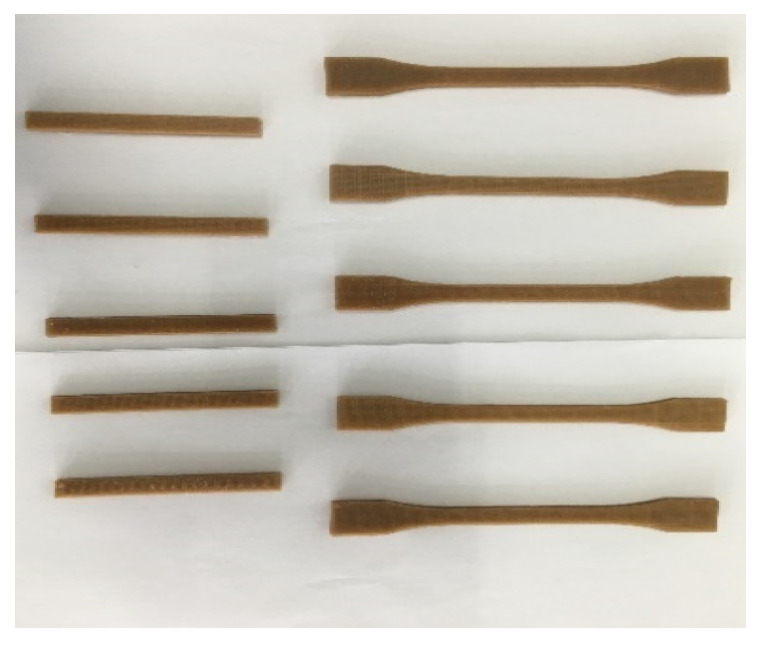
3D printed PLA/ROA composite specimens.

A MOSHU S108 desktop-level 3D printer from Hangzhou Shining 3D Technology Co. Ltd. (Hangzhou, China) was used and equipped with a 0.4 mm nozzle. The sample model file (STL file) for the test was computer-aided designed and was then sliced and transformed into G-code. The printing parameters were set using the same software. Using PLA/ROA composite filament as the printing material, the samples (Figure 2) were then FDM 3D printed. For comparison, the printing parameters concerned included the printing temperatures of 200, 210, and 220 °C, the printing speeds of 50, 60, and 70 mm/s, the filling densities of 60, 80, and 100%, the printing layer thicknesses of 0.1 and 0.2 mm, and the deposition angles of 0, 45, and 90°.

### 2.4. Measurement and Characterization 

#### 2.4.1. Mechanical Testing

Tensile and flexural specimens were conditioned at room temperature (23 °C and 45% relative humidity) and characterized using a universal machine (E44.304, MTS Industrial Systems (China) Co. Ltd., Shenzhen, China) with a load cell capacity of 20kN according to the ASTM D 638-2010 standard and the ASTM D790-2010 standard at a crosshead speed of 10mm/min and 5mm/min, respectively. At least five specimens were analyzed for each batch, and average values of modulus and strength at max were calculated.

#### 2.4.2. Morphological Observation

The cross-sectional morphologies of the specimens obtained at different printing parameters after flexural testing were observed using an optical microscope (BX51, Olympus Corp., Tokyo, Japan). The magnification is 100 times.

The cross-sectional morphology of the specimen obtained at the optimum printing conditions after flexural testing were observed using a scanning electron microscope (SEM) (SU8010, HITACHI Corporation, Tokyo, Japan). The sample was coated with a thin layer of gold to avoid sample charging during imaging and then examined under SEM. The microstructure of the sample was magnified and digitally recorded. 

#### 2.4.3. Melt Flow Rate (MFR)

The MFR of the PLA/ROA composite specimens printed at the optimum conditions was examined according to the Chinese national standard GB/T 3682-2000 to characterize the flowability of the samples. The samples were weighed and put into the melt index meter (XNR-400, Chengde Jinhe Instrument Manufacturing Co. Ltd., Chengde, China). The MFR measurements were carried out at 180 °C and 1.26 kgf. 

#### 2.4.4. Thermogravimetric Analysis (TGA)

A thermal gravimetric analyzer (TG 209F1, NETZSCH-Gerätebau GmbH, Selb, Germany) was used to evaluate the thermal stability of FDM 3D-printed PLA/ROA samples. Approximately 5 mg~12 mg of each sample was weighed into an alumina ceramic crucible, and placed onto the balance of the TGA instrument, then measured from 20 °C up to 550 °C under nitrogen atmosphere at a 20 K/min heating rate. Before use, the equipment had undergone three times nitrogen gas evacuation.

#### 2.4.5. Differential Scanning Calorimetry

A differential scanning calorimetry (DSC214, NETZSCH-Gerätebau GmbH, Selb, Germany) was used to investigate the melting and crystallization behavior. An amount of 10 ± 1 mg of each sample was weighed in hermetically sealed aluminum pans, sample temperature was first increased from the ambient temperature to 220 °C at a heating rate of 10 °C/min and held in an isothermal state for 5 min, to eliminate thermal history, residual moisture, and voids. Then, the sample was cooled down to room temperature at 10 °C/min, and finally reheated to 220 °C at 10 °C/min, using an empty pan as a reference. The cold-crystallization temperature (Tcc) and the melt temperature (Tm) were determined from the second heating curves. The glassy transition temperatures and heat capacities were calculated via the NETZSCH analysis software. The degree of crystallinity was calculated using the following equation:

(1) $$x_{c}=\frac{\left|\Delta H_{m}+\Delta H_{cc}\right|}{\omega \Delta H^{*}}\times 100\%$$

where $x_{c}$ refers to the degree of crystallinity of the sample, ω refers to the mass fraction of PLA matrix in the composite, $\Delta H_{m}$ refers to the melting enthalpy change (J/g), $\Delta H_{\mathrm{cc}}$ refers to the enthalpy change of cold crystallization (J/g), and $\Delta H^{*}$ refers to the melting enthalpy of 100% crystallization of PLA (J/g), which was 93.6 J/g according to the literature [17]

#### 2.4.6. Wettability 

The contact angles of the FDM 3D-printed PLA/ROA composite specimens were measured with a contact angle instrument (DSA100; KRÜSS GmbH, Borsteler Chaussee, Germany) using a distilled water drop at room temperature. A 5 µL droplet of distilled water was dropped onto the specimen surface and kept for 15 s, and then the contact angles from the images were measured at different points; 10 specimens were used for each sample. 

## 3. Results and Discussion 

### 3.1. Mechanical Properties 

To understand the effect of the printing process on the mechanical properties of PLA/ROA composites, experiments were carried out on the printed specimens with different printing temperatures, printing speeds, filling densities, layer thicknesses, and deposition angles. The mechanical properties of the specimens were gathered in Table 1. 

For FDM 3D technology, the printing temperature is an essential parameter and should be controlled properly. When the printing temperature is too low, the filament starts to clog within the extruder, whereas when it is too high, it starts leaking during printing [18]. In this study, the printing temperature was set as 200 °C, 210 °C, and 220 °C, respectively. The results showed that the printing temperatures significantly affected the mechanical properties of the 3D-printed PLA/ROA composites (referring to the samples No. 1, 2, and 3 in Table 1). The highest mechanical properties were found in the specimen with printing temperature of 220 °C (sample No. 3), followed by 210 °C (sample No. 2), and 200 °C (sample No. 1), respectively. For example, the tensile strength of the sample No. 3 was found to be 23.51 MPa, while they were found to be 20.28 MPa and 17.46 MPa for the samples No. 2 and No. 1, respectively. Similar results were found in the tensile modulus and flexural properties. It can be concluded that a higher printing temperature is good for the improvement of mechanical properties within a certain temperature range; the reason may be that an increased printing temperature can promote the flowability of the melt. The printed specimen may have a more homogeneous and compact structure, and at the same time, the melt strength can also be enhanced. 

**Figure 3:**
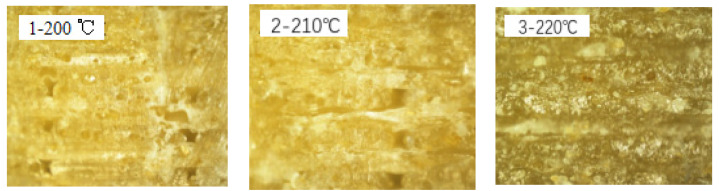
Optical microscopy images (×100 times) of specimen cross section at different printing temperatures.

Cross-sections of the printed samples obtained at different printing temperatures were placed under an optical microscope, and images taken at 100 times magnification were presented in Figure 3 to evaluate the inter-strand and inter-layer coalescence. The results showed that the sample printed at 220 °C (sample No. 3) had the best overall coalescence, while there was a gradual loss of the same with decreasing temperature, proving that the printing temperature variation had a direct effect on the meso-structural quality of the printed material. The results of the inter-strand and inter-layer coalescence were positively correlated with those of the mechanical properties. 

**Figure 4:**
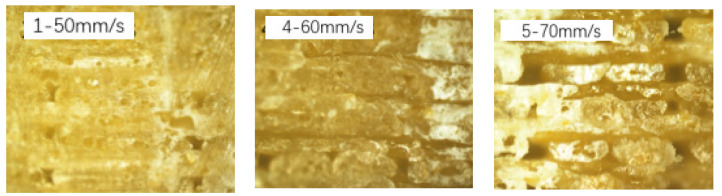
Optical microscopy images (×100 times) of specimen cross section at different printing speeds.

The results of samples No. 1, 4, and 5 in Table 1 demonstrated the influences of the printing speed on the mechanical properties of the 3D-printed PLA/ROA composites. The fractural morphologies of PLA/ROA composites obtained at different printing speeds observed by optical microscope were shown in Figure 4. The tensile strength, tensile modulus, flexural strength, and flexural modulus of the sample with a printing speed of 50 mm/s (sample No. 1) were found to be 17.46 MPa, 234.27 MPa, 97.60 MPa, and 16,406.04 MPa respectively, however, those were 14.36 MPa, 162.21 MPa, 62.22 MPa, and 13,545.13 MPa for the sample with a printing speed of 60 mm/s (sample No. 4), and 13.25 MPa, 154.62 MPa, 60.43 MPa, and 10,820.75 MPa for the sample with a printing speed of 70 mm/s (sample No. 5). Overall, these mechanical properties of the composites decreased as the printing speed increased. This was due to the poorer precision and uneven bonding between the upper and lower layers with the increased printing speed as shown in Figure 4, inducing the weakened interfacial adhesion between different printing layers, and even the occurrence of the cavities and delamination at the interface between different layers. The reduction in mechanical properties of 3D-printed samples with the increased printing speed was also reported by Zhu et al. [19]. 

Rebenaque et al. found that, when increasing the filling density, the maximum strength to bending of FDM 3D-printed PLA specimens increases considerably [20] The similar conclusions can be drawn in this study. For FDM 3D printed PLA/ROA composite specimen, as was shown in Table 1, the tensile strength, tensile modulus, flexural strength and flexural modulus of the sample with a filling density of 100% (sample No. 1) were 17.46 MPa, 234.27 MPa, 97.60 MPa, and 16,406.64 MPa, while those of the sample with a density of 80% (sample No. 7) were 16.53 MPa, 215.26 MPa, 94.31 MPa, and 15,878.05 MPa, which were 5.33%, 8.11%, 3.37%, and 3.22% smaller than those of sample No. 1, respectively. For the sample with a filling density of 60% (sample No. 6), its strengths and moduli were decreased furthermore, in this situation, its tensile strength, tensile modulus, flexural strength, and flexural modulus were only 84.76%, 88.24%, 89.38%, and 87.61% those of sample No. 1, respectively. 

**Figure 5:**
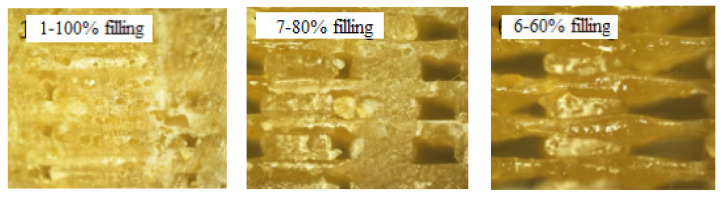
Optical microscopy images (×100 times) of specimen cross section at different filling densities.

Figure 5 showed the optical microscopy images of the specimen cross section at different filling densities. Obviously, filling density had a great relationship with the compactness of filling in the shell of 3D printing parts, which is well in accordance with the result reported in the literature [19]. With the increase of the filling density, the internal structure of the printed sample became more compact, and fewer internal defects existed; the mechanical properties of the sample would thus be accordingly improved. The decrease in mechanical properties of FDM 3D printed specimens with the reduction of filling density may be due to the poorer adhesion between neighboring layers because of the lack of bonding points induced by lower filling density. 

**Figure 6:**
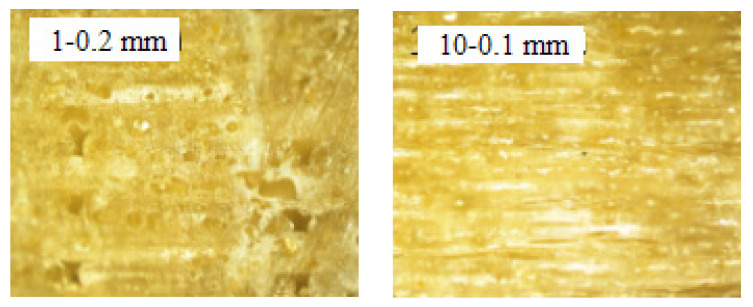
Optical microscopy images (×100 times) of specimen cross section at different printing layers.

Effects of printing layer thickness on mechanical properties including tensile strength, tensile modulus, flexural strength, and flexural modulus of FDM 3D printed PLA/ROA composite parts were shown in Table 1, labelled as the samples No. 1 and 10. It was observed that all the tensile and flexural strength and modulus decreased with the increased printing thickness, and the printing thickness greatly affected the mechanical properties of the 3D printed PLA/ROA composite specimens, which was consistent with those of previous studies [2,19]. Figure 6 illustrated the fractural morphologies of PLA/ROA composites obtained at different printing layers observed by optical microscope. When the printing thickness decreased from 0.2mm to 0.1mm, the number of layers in the specimen increased when the thickness of all the samples was kept constant, and the deposited filaments were squeezed more heavily by the nozzle so that the interlamination were tightly combined. This may result in higher integrity that in turn will improve the mechanical properties of the specimens. In addition, a smaller layer thickness may lead to smaller airgap to material ratio, in which the breaking point is reached at higher loads [17]. 

The mechanical properties of the 3D-printed PLA/ROA composites with different deposition angles were exampled as samples No. 1, 8, and 9 in Table 1. It can be found that the sample with the deposition angle of 0° (sample No. 8) had the greatest tensile and flexural strengths and moduli, its tensile strength of 22.63MPa was greater by 29.61% and 30.36%, respectively, than those of the samples with the deposition angle of 45° (sample No. 1) and 90° (sample No. 9). Similarly, the tensile modulus was greater by 24.98% and 32.41%, the flexural strength by 5.37% and 14.74%, and the flexural modulus by 3.08% and 6.30%, respectively. After comparison, we found that the effect of the deposition angle on the tensile properties of the printed specimens were greater than on the flexural properties.

Similar mechanical properties results because of the different deposition angles were reported by Marie-Joo et al. [4] and Duigou et al. [5] for 3D printed rice husk or wood/PLA rectangular beam. They suggested that the mechanical properties differed from the printing directions were due to the lack of interlayer interactions.

**Figure 7:**
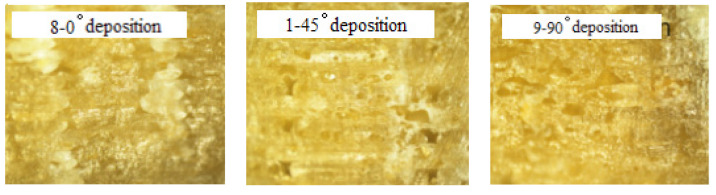
Optical microscopy images (×100 times) of specimen cross section at different deposition angles.

The fractural morphologies of PLA/ROA composites obtained with different deposition angles were shown in Figure 7, it indicated that the structure of the specimen deposited at 0° angle was really the most homogeneous among the three samples. 

**Figure 8:**
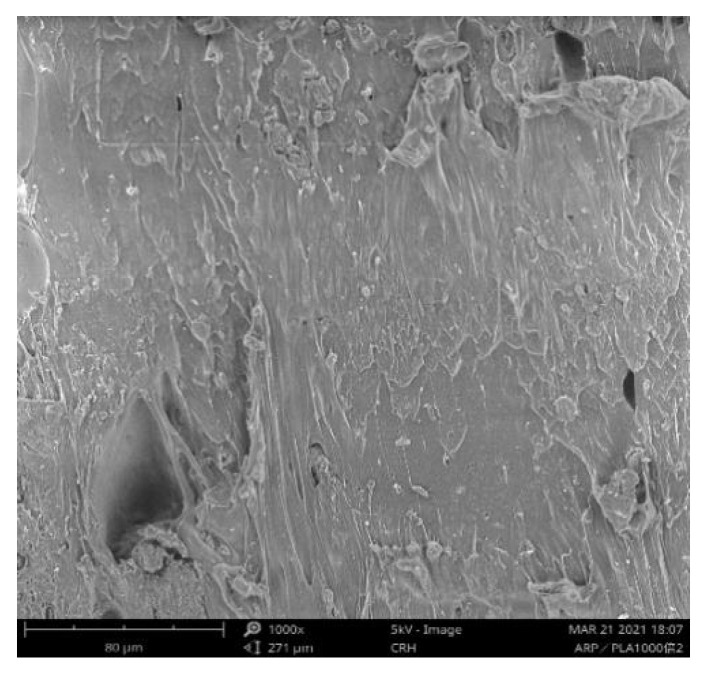
SEM micrograph of PLA/ROA composite specimen.

From above discussions, it was learned that the optimum printing parameters were the printing temperature of 220 °C, the printing speed of 50 mm/s, the filling density of 100%, the printing layer thickness of 0.1mm, and the deposition angle of 0°. Figure 8 demonstrated the SEM images of the cross-sectional morphology of the sample prepared at the optimum conditions, it was clear that a good miscibility existed between various components. Generally, all the FDM 3D-printed PLA/ROA composite specimens had adequate comprehensive mechanical properties; though different from one another because of the varying printing parameters, they could be fabricated into a large variety of items such as teaching models, lamp holders, and craft boxes. 

### 3.2. Flow Ability 

**Figure 9:**
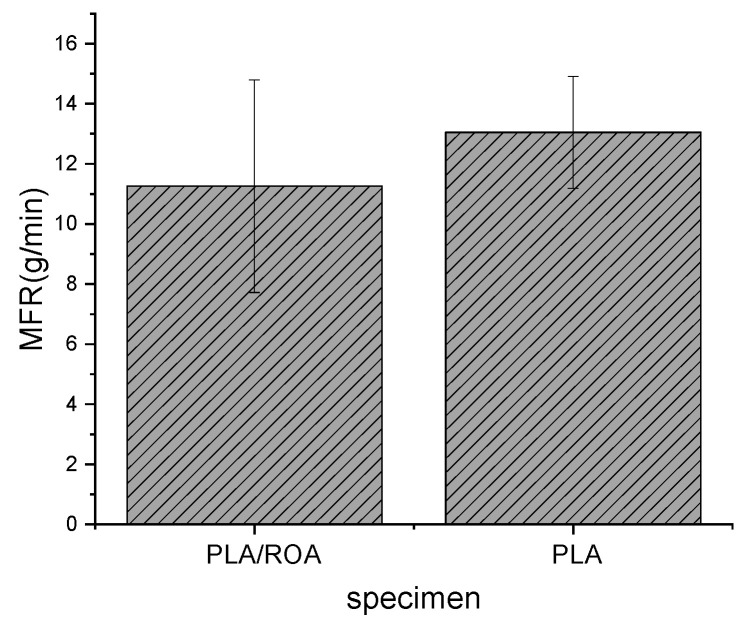
MFR of PLA/ROA composite specimen and pure PLA.

In order to evaluate the processing properties and printability of FDM 3D printed PLA/ROA composite specimen, MFR of the sample prepared at the optimum conditions was measured. The result was shown in Figure 9. Meanwhile, the MFR of pure PLA was also illustrated for comparison. Incorporation of ROA reduced the MFR from 13.05 g/10 min for PLA to 11.26 g/10 min for the composite, which was because the natural fiber had great effects on the movement of the molecular chains of PLA. When PLA was complexed with ROA, it turned to be more difficult for the movement of the molecular chains, and as a result, the flowability of the resin became poorer. Even so, the MFR of PLA/ROA composite was still high enough and could meet well the requirement for processing.

### 3.3. Thermal Stability 

**Figure 10:**
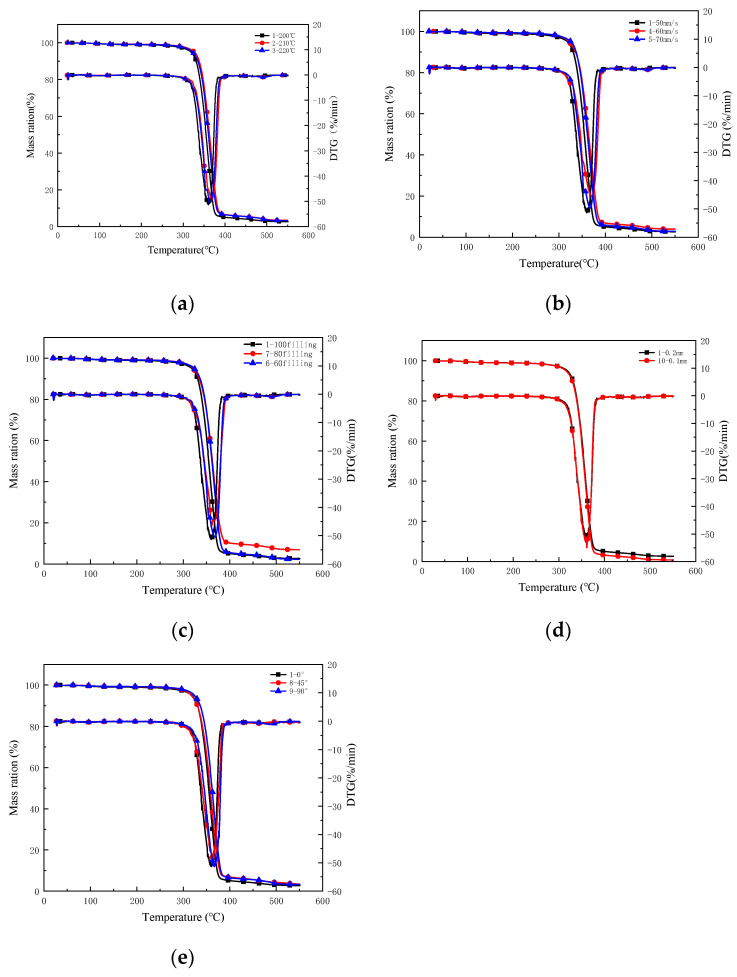
The mass loss curves of different printing parameters (**a**) printing temperature; (**b**) printing speed; (**c**) filling density; (**d**) printing layer thickness; (**e**) deposition angle.

Thermal stability of FDM-3D printed PLA/ROA composite specimen was studied by thermo-gravimetric analysis. Figure 10a–e showed the weight loss over time curves and their corresponding derivatives of the specimens at different printing conditions. It can be found that each sample had a mass loss of around 5% at around 100 °C, which was due to the evaporation of moisture and the release of the small molecular substances in the sample. The major decomposition for each specimen all happened between 330 °C and 410 °C. 

To compare the relative thermal stability between samples, the initial decomposition temperature (T_i_) and the temperature at which the decomposition happened the fastest (T_p_) in the major decomposition stage were chosen. Meanwhile, the mass residues after the major decomposition (w) were also recorded as a reference. The results were listed in Table 2.

It can be seen from Figure 10a and Table 2 that the T_i_ value of the printed specimens increased gradually with the increase of the printing temperature, the T_i_ value of the specimen printed at 220 °C (sample No. 3) was 339.4 °C, which was 2.3 °C and 2.5 °C higher than those of the specimens printed at 210 °C (sample No. 2) and 200 °C (sample No. 1), respectively. The T_p_ value of sample No. 2 was a little higher than that of sample No. 1 by 1.2 °C, however, the T_p_ value of sample No. 3 was 369.1 °C, obviously increased from those of the former two specimens. Data obtained from the DTG curves corroborated the increasing thermal stability of the composites at higher printing temperatures.

From Figure 10b and Table 2, the printed specimen at a printing speed of 50 mm/s (sample No. 1) exhibited a T_i_ value of 336.9 °C and T_p_ value of 363.3 °C. When the printing speed was increased to 60 mm/s (sample No. 4), the T_i_ and T_p_ values were lowered to 336.1 °C and 362.3 °C, and for the specimens at a printing speed of 70 mm/s (sample No. 5), the values were further reduced to 334.5 °C and 361.1 °C, respectively. This suggested that the PLA/ROA composite at a smaller printing speed was more thermally stable than the materials at a greater printing speed. 

Results of the TGA tests conducted on the printed specimens with different filling densities were presented in Figure 10c. It was observed from Figure 10c and Table 2 that the specimen with a filling density of 100% (sample No. 1) had the highest T_i_ value of 336.9 °C and T_p_ value of 363.3 °C, which were 2.0 °C and 1.4 °C higher than those of the specimens with a filling density of 80% (sample No. 7) and 5.2 °C and 3.2 °C higher than those of the specimens with a filling density of 60% (sample No. 6), indicating that increasing the filling density was good for the improvement of the thermal stability of the FDM 3D printed specimens. This was probably caused by the difficult movement of the molecular chains of the sample due to the greater filling density.

The TGA and DTG curves of the printed specimens with different printing layer thickness were shown in Figure 10d. The major decomposition of each sample took place at temperature higher than 330 °C. The major decomposition of the specimen with a printing layer thickness of 0.2 mm (sample No. 1) started at 336.9 °C, and the fastest decomposition happened at 363.3 °C, while the corresponding temperatures for the specimen with a printing layer thickness of 0.1 mm (sample No. 10) were 339.6 °C and 369.1 °C. The results showed that the decrease of the printing layer thickness resulted in an increase of thermal stability. 

Figure 10e showed thermal degradation curves of the 3D printed PLA/ROA composite specimens at different deposition angles (samples No. 1, 8, and 9). It was noticed that there were very little differences in both the T_i_ and T_p_ values among the specimens, indicating that the deposition angle had little effect on the thermal stability of the printed PLA/ROA specimen. 

### 3.4. Melting and Crystallization Behavior 

**Figure 11:**
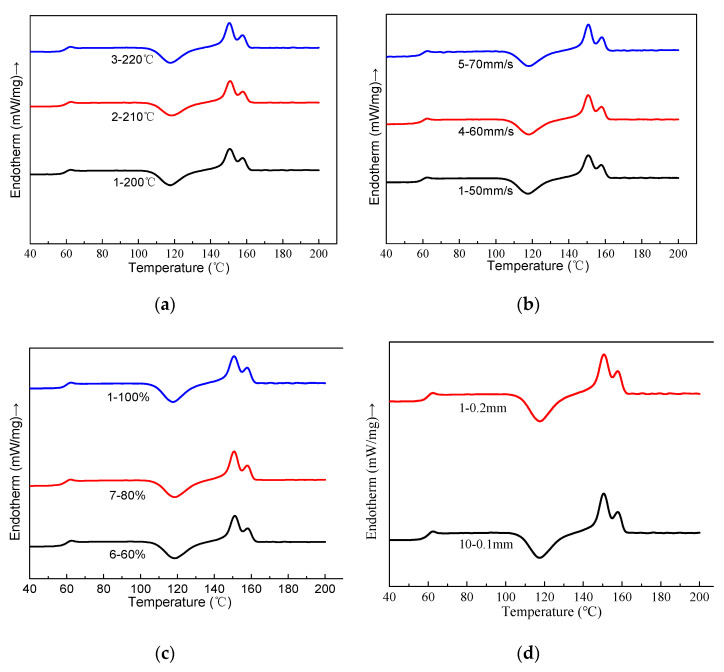

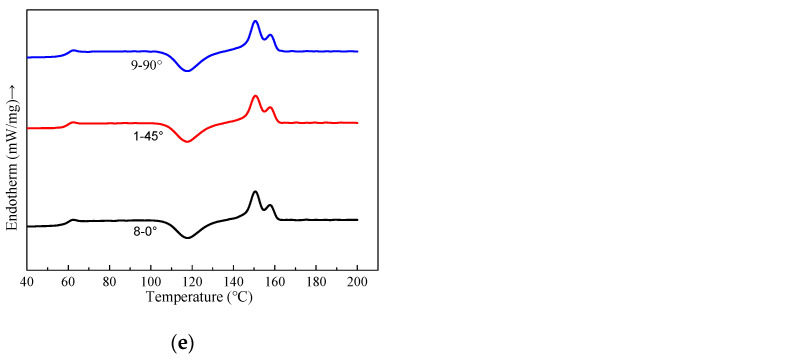
The secondary heating curves of specimen at different printing parameters: (**a**) printing temperature; (**b**) printing speed; (**c**) filling density; (**d**) printing layer thickness; (**e**) deposition angle.

Figure 11 showed the melting and crystallization transition curves of the printed PLA/ROA composite specimens. The results derived from Figure 11 were listed in Table 3. It was found that the melting endotherm of each sample displayed two melting peaks at around 151 and 158 °C. The double-melting behavior was probably linked to the formation of different crystal structures, and the lower one corresponded to the melting of crystals with an imperfect structure. Similar phenomena were also described by Hao Liu et al. [11] and Guim et al. [21]. Endotherms in Figure 11 indicated that PLA/ROA composite specimens had well-defined glassy transition peaks centered at around 62.5 °C. The difference of the glassy transition temperature (T_g_) among the printed specimens obtained at various printing parameters was actually very tiny (Table 3). In addition, a big cold crystallization peak appeared during the second heating process. This crystallization behavior was attributed to too slow crystallization rate of pure PLA to well organize its molecular chains timely [11]. 

Exotherms shown in Figure 11 and the corresponding results listed in Table 3 indicated that the change of the printing parameters had also little effect on the cold crystallization temperature (T_cc_) value of the printed PLA/ROA composite specimen; the T_cc_ value of each specimen was generally at around 118 °C. Additionally, the T_m_ value of each specimen was also close to each other. According to the results of the DSC analysis, variation of printing parameter had little effects on the melting properties of the FDM 3D printed specimens.

### 3.5. Wettability

**Figure 12:**
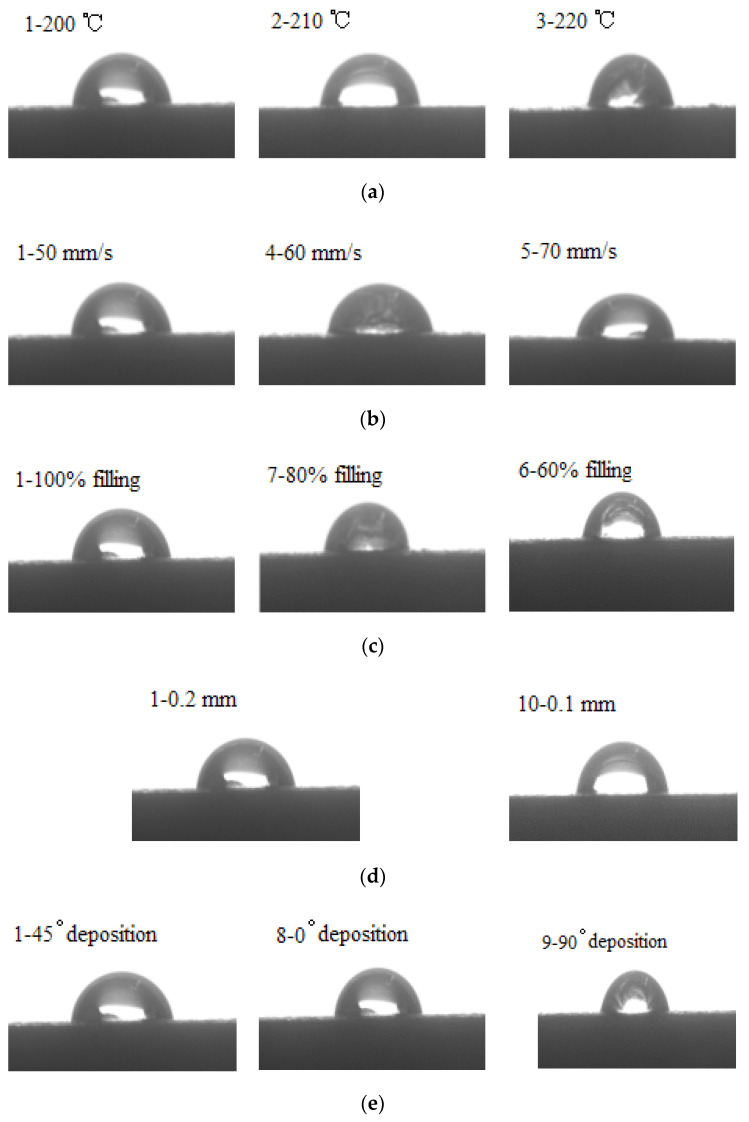
Contact angles of deionized water on surfaces at different printing parameters:(**a**) printing temperature; (**b**) printing speed; (**c**) filling density; (**d**) printing layer thickness; (**e**) deposition angle.

The shapes of water droplets on the surfaces of the printed specimens were shown in Figure 12a–e. The measured averaged contact angles were summarized in Table 4. The results showed that the contact angle values of the specimens increased slightly with the increase of the printing temperature or the filling density, while they decreased with the increase of the printing speed, the deposition angle, or the printing thickness. Among all the printing parameters, the printing speed had the greatest effects on the contact angle values, the specimen printed at a speed of 70 mm/s (sample No. 5) had the smallest contact angle of 70.5°, which was 3.2° and 7.7° lower than those on the specimens printed at a speed of 60 mm/s (sample No. 4) and 50 mm/s (sample No. 1), respectively. The decrease in the contact angle value was very obvious, which meant that the wettability of the specimens decreased with the increasing printing speed significantly. Though the other parameters all had effects on the wettability of the specimens, the differences in the contact angle values among these specimens did not differ from one another greatly; that is to say, the printing parameters except for the printing speed had tiny effects on the wettability of the printed specimens. 

As a natural fiber, ROA was composed of cellulose, hemicellulose, lignin, and some other components. The existence of a large amount of the polar groups, such as hydroxyl in its structure, makes ROA hydrophilic. When PLA is complexed with ROA, the hydrophilicity of the material will be improved, though PLA is hydrophobic by itself. It can be found that all the contact angle values of the printed PLA/ROA composite samples fell into the range between 70 to 81°, much less than 90°, indicating that wetting of the surface was favorable [22].

## 4. Conclusions

In this study, 3D-printed PLA/ROA biomass composites were manufactured using FDM 3D printing technology, and the effects of printing parameters on the properties of PLA/ROA composite specimens were investigated. The following conclusions can be drawn from the research:

(1) The mechanical properties could be improved when the printing temperature was increased from 200 °C to 220 °C or the filling density was increased from 60% to 100%; However, the properties would be decreased when the printing speed was increased from 50 mm/s to 70 mm/s, or the printing thickness was increased from 0.1 mm to 0.2 mm. When the effects of deposition angles on the mechanical properties were concerned, the specimen at a printing angle of 0° has the greatest mechanical properties, which were 22.63 MPa, 292.80 MPa, 102.84 MPa, and 16,910.96 MPa respectively for the tensile strength, tensile modulus, flexural strength, and flexural modulus.
(2) The thermal stability of the FDM 3D printed PLA/ROA composite specimens could be improved either by increasing the printing temperature and the filling density or by reducing the printing speed and the printing thickness. The specimen printed at a deposition angle of 0° was more thermally stable than those printed at a deposition angle of 45° or 90°, but not remarkably.
(3) The melting and crystallization behavior of the FDM 3D-printed PLA/ROA composite specimens was little affected by the printing parameters. The glassy transition temperature of each specimen was all around 62.5 °C, the cold crystallization temperature of each specimen was generally at around 118 °C, and the melting endotherm of each sample displayed two melting peaks at around 151 and 158 °C.
(4) All the printed specimens are hydrophilic; the wettability of the specimens would be improved with the increase of the printing speed, the printing thickness, and the deposition angle, or the decrease of the printing temperature and the filling density. Among all the parameters, the printing speed could affect the wettability of the sample the most significantly.

Though the printing parameters have effects on its mechanical properties, thermal stability, and wettability, the PLA/ROA composite specimen has proper comprehensive properties and can also be printed smoothly. It can be used as a new kind of filament material for FDM 3D printing.

## Figures and Tables

**Table 1:** Mechanical properties of 3D-printed PLA/ROA composite samples at different printing conditions.

Sample No.	Filling Density (%)	Deposition Angle (°)	Printing Speed (mm/s)	Printing Temperature (°C)	Printing Layer Thickness (mm)	Flexural Strength (MPa)	Flexural Modulus (MPa)	Tensile Strength (MPa)	Tensile Modulus (MPa)
1	100	45	50	200	0.2	97.60 ± 4.25	16,406.04 ± 553.84	17.46 ± 1.28	234.27 ± 12.88
2	100	45	50	210	0.2	100.23 ± 9.21	16,799.14 ± 656.54	20.28 ± 2.46	265.92 ± 11.86
3	100	45	50	220	0.2	120.93 ± 9.68	17,333.35 ± 486.42	23.51 ± 2.88	316.75 ± 23.58
4	100	45	60	200	0.2	62.22 ± 6.53	13,545.13 ± 638.62	14.36 ± 1.12	162.21 ± 13.57
5	100	45	70	200	0.2	60.43 ± 5.46	10,820.75 ± 711.33	13.25 ± 1.02	154.62 ± 10.67
6	60	45	50	200	0.2	87.24 ± 5.96	14,373.35 ± 567.46	14.80 ± 1.86	206.73 ± 19.01
7	80	45	50	200	0.2	94.31 ± 8.62	15,878.05 ± 609.02	16.53 ± 1.08	215.26 ± 15.25
8	100	0	50	200	0.2	102.84 ± 9.22	16,910.96 ± 710.87	22.63 ± 2.12	292.80 ± 17.92
9	100	90	50	200	0.2	89.63 ± 7.03	15,908.66 ± 822.31	17.36 ± 2.56	221.13 ± 16.43
10	100	45	50	200	0.1	107.46 ± 8.88	17,547.12 ± 803.48	20.71 ± 2.62	279.70 ± 20.08

**Table 2:** TGA and DTGA data of specimens at different printing parameters.

Sample No.	Ti/°C	T_p_/°C	W/%
1	336.9	363.3	2.35
2	337.1	364.5	3.13
3	339.4	369.1	3.27
4	336.1	362.3	1.64
5	334.5	361.1	4.75
6	331.7	360.1	2.69
7	334.9	361.9	2.77
8	337.8	364.1	5.15
9	337.6	363.8	2.43
10	339.6	369.1	3.44

**Table 3:** DSC thermal information table of specimen at different printing parameters.

Sample No.	T_g_/°C	T_cc_/°C	T_m_/°C	ΔH_cc_/(J/g)	ΔH_m_/(J/g)	Χ_c_/%
1	62.4	117.6	150.7	−26.30	27.90	1.9
2	62.6	118.1	150.9	−23.92	26.60	3.2
3	62.5	117.8	150.5	−27.58	29.19	2.0
4	62.5	117.9	150.7	−25.61	27.37	2.1
5	62.5	118.2	150.8	−24.54	28.11	4.3
6	62.7	118.8	151.1	−25.07	25.78	0.8
7	62.1	118.7	150.8	−26.27	26.03	0.3
8	62.4	117.9	150.6	−24.65	27.32	3.2
9	62.6	117.6	150.6	−28.61	30.56	2.4
10	62.5	117.5	150.6	−24.31	26.64	2.8

**Table 4:** Contact angles of deionized water on surfaces at different printing parameters.

Sample No.	1	2	3	4	5	6	7	8	9	10
Contact angle/°	78.2 ± 0.5	79.1 ± 0.4	80.0 ± 0.6	73.7 ± 0.5	70.5 ± 0.3	76.6 ± 0.6	77.4 ± 0.5	79.3 ± 0.5	78.0 ± 0.8	80.1 ± 0.6

## Data Availability

Not applicable.

## Sample Availability

Samples of the compounds are not available from the authors.
